# Gestational Weight Gain Following Metabolic Bariatric Surgery: A Scoping Review

**DOI:** 10.3390/nu16152516

**Published:** 2024-08-01

**Authors:** Ellen Deleus, Matthias Lannoo, Dries Ceulemans, Roland Devlieger, Bart Van der Schueren, Katrien Benhalima

**Affiliations:** 1 Department of Chronic Diseases and Metabolism, KU Leuven, 3000 Leuven, Belgium; matthias.lannoo@uzleuven.be (M.L.); bart.vanderschueren@uzleuven.be (B.V.d.S.); katrien.benhalima@uzleuven.be (K.B.); 2 Department of Abdominal Surgery, University Hospitals Leuven, 3000 Leuven, Belgium; 3 Department of Development and Regeneration, KU Leuven, 3000 Leuven, Belgium; dries.ceulemans@uzleuven.be (D.C.); roland.devlieger@uzleuven.be (R.D.); 4 Department of Obstetrics and Gynecology, University Hospitals Leuven, 3000 Leuven, Belgium; 5 Department of Endocrinology, University Hospitals Leuven, 3000 Leuven, Belgium

**Keywords:** gestational weight gain, metabolic bariatric surgery, sleeve gastrectomy, Roux-en-Y gastric bypass, birth weight, small-for-gestational age

## Abstract

Metabolic bariatric surgery remains the most effective and durable treatment for severe obesity. Women of reproductive age represent the largest demographic group undergoing these procedures. Metabolic bariatric surgery can have both beneficial and adverse effects on pregnancy outcomes. One of the most common adverse effects is fetal growth restriction. To mitigate these adverse effects, it is crucial to explore lifestyle modifications aimed at promoting a healthy pregnancy. Modifiable factors during pregnancy after metabolic bariatric surgery include the amount of gestational weight gain. The aim of this comprehensive review is to provide an overview of what is known about gestational weight gain in pregnancy after bariatric metabolic surgery. This review is focused on the two most performed procedures: sleeve gastrectomy and Roux-en-Y gastric bypass.

## 1. Introduction

Pregnancies in women with a history of metabolic bariatric surgery are becoming increasingly prevalent. Surgically induced metabolic changes benefit mother and child but can also lead to certain adverse pregnancy outcomes [1]. Adverse pregnancy outcomes include shorter gestation and increased risk of small-for-gestational-age (SGA) infants [2]. The etiopathogenesis of these adverse effects remains largely unknown. In 2009, the Institute of Medicine [currently known as the US National Academy of Medicine (NAM)], published ranges for optimal gestational weight gain (GWG) in pregnancies, depending on pre-pregnancy body mass index (BMI) [3]; see Table 1.

It is unknown whether these proposed ranges are also appropriate for a population of women who underwent metabolic bariatric surgery prior to pregnancy. Additionally, these recommendations are expressed in absolute kilograms rather than as a percentage of total weight. However, most guidelines for GWG in pregnancy after metabolic bariatric surgery rely entirely on NAM recommendations [1,4,5]. Furthermore, most societies recommend monitoring for nutritional deficiencies and routinely implementing dietary counseling during pregnancy in order to maintain active surveillance of nutritional status; see Table 2.

The primary objective of this scoping review was to compare GWG between pregnancies following metabolic bariatric surgery and control groups. We searched for studies on sleeve gastrectomy and/or Roux-en-Y gastric bypass (RYGB) since these are the two most performed procedures worldwide. This was carried out to exclude older procedures with distinct mechanisms of action, such as adjustable gastric banding and biliopancreatic diversion. Secondary objectives included comparing GWG between sleeve gastrectomy and RYGB procedures, exploring the impact of surgery-to-conception interval on GWG, investigating the impact of GWG on birth weight, and finally looking for dietary interventions to optimize GWG after metabolic bariatric surgery. 

## 2. Methods

We searched PubMed (including MEDLINE), Embase, Web of Science Core Collection, Scopus, and CENTRAL (Cochrane Library) from inception to 15 May 2024. We did not include preprints or clinical trial registries in the search. The search strategy was based on the following two concepts “bariatric surgery” and “gestational weight gain”. A detailed overview of all search strings can be found in Supplementary File S1. The following inclusion criteria were applied: observational or interventional studies reporting GWG following sleeve gastrectomy or RYGB with a singleton pregnancy. If different types of metabolic bariatric surgery were included as exposures, we required the ability to extract data specifically for sleeve gastrectomy and/or RYGB. If this was not possible, we mandated that at least 95% of participants underwent either sleeve gastrectomy, RYGB, or both. For the primary analysis, we included only original cohort studies with a non-surgical control group, either matched or unmatched. Reference lists of key studies and reviews were manually screened to identify additional relevant articles. No limitations regarding language were applied. For the secondary objectives, we included observational or interventional studies that either lacked a non-surgical control group or involved mixed exposures, encompassing different types of surgery. We also included review or guideline manuscripts. Furthermore, since this is a scoping review, not all studies are discussed. 

## 3. Results of the Systematic Search

**Figure 1:**
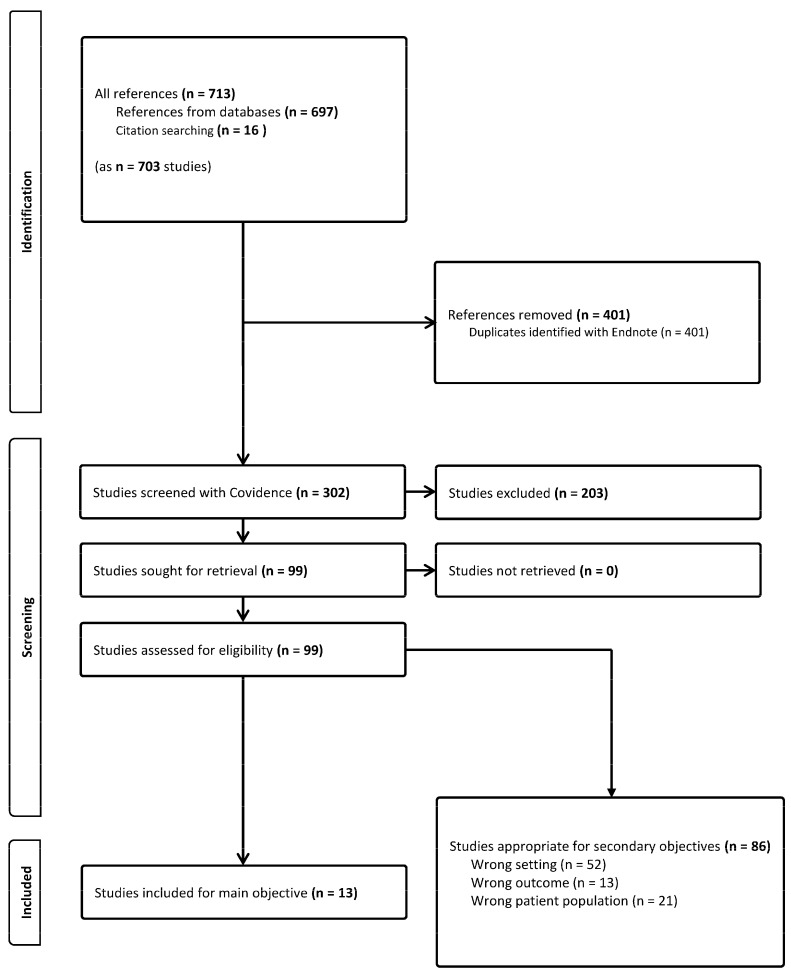
PRISMA flow chart.

We identified 13 original articles fitting the main objective of the systematic search. In 12 studies, the size of the metabolic bariatric surgery group ranged from 23 up to 151 pregnancies, and the size of the control group varied from 23 up to 311 pregnancies. There was one exceptionally large retrospective population study with 670 pregnancies after metabolic bariatric surgery, compared to 627,023 control pregnancies [7]. An overview of the PRISMA flow chart can be found in Figure 1. All studies we identified were observational cohort studies. The studies investigated the effects of metabolic bariatric surgery by comparing gestational, maternal, and neonatal outcomes with a control group that had not undergone such surgery. An overview of all articles can be found in Supplementary File S2 [7,8,9,10,11,12,13,14,15,16,17,18,19]. Whenever we found a secondary study based on (part of) the same patient population, we added these to the overview following the main publication [20,21,22]. We identified a retrospective study where overlap in patient population from previous authors was described; as such, the main paper was analyzed [16], whilst the other three were not included [23,24,25]. All papers we analyzed were published within the last 10 years, reflecting the current relevance of the topic. 

Furthermore, we identified 86 studies not fitting the main objective of the systematic search but providing insights into secondary questions for this review. These studies either did not have a control group (wrong setting), did not report the amount of GWG (wrong outcome), or had a patient population that was too heterogenous (wrong population). We will discuss certain studies mentioned here, because of their importance in providing further insights. 

## 4. Gestational Weight Gain Following Metabolic Bariatric Surgery: Does It Differ from Pregnancies in the Non-Surgical Population?

There is a worrying trend for excessive GWG in women with overweight or obesity, according to NAM recommendations [3,26]; see Table 1. Furthermore, several experts signal that especially in this population, excessive GWG can have negative consequences on pregnancy outcomes [27].

Regarding pregnancy following metabolic bariatric surgery, we identified four cohort studies reporting on GWG according to NAM [9,11,12,19]; see Table 3. These research groups compared the adequacy of GWG in pregnancies after RYGB compared to non-surgical controls. First, Stentebjerg et al. [9] conducted a prospective cohort study, where 23 pregnancies in women after RYGB were compared to 23 pre-pregnancy BMI-matched control pregnancies. The main aim of the study was to investigate glucose profiles measured by continuous glucose monitoring. After RYGB, most women exhibited non-appropriate weight gain, both excessive (44%), as well as insufficient (39%), while most women in the control group also had excessive weight gain (57%). Second, a retrospective cohort study conducted by Hammeken et al. [12] looked at pregnancy outcomes after RYGB (*n* = 151) versus non-operated controls (*n* = 151), matched based on their pre-pregnancy BMI, among other factors. The study showed that the majority of participants in both groups had non-appropriate weight gain, mostly because of excessive weight gain. Unfortunately, there was a significant amount of missing data: 19.2% and 13.9% in the metabolic bariatric surgery and control groups, respectively. A recent longitudinal observational study performed by Iacovou et al. [11] examined 79 pregnancies after sleeve gastrectomy or RYGB, compared to a non-surgical control group with 100 participants. The primary matching was conducted based on pre-pregnancy BMI, among other factors. As mentioned in the Methods section, we excluded data on outcomes after the gastric band for this analysis. The mean BMI in both the surgical and control groups was in the range of obesity. Findings regarding GWG were similar to the previous studies: in all groups, more than half of pregnant women had non-appropriate weight gain, mostly because of excessive GWG. The proportion of pregnancies with excessive GWG was 40.8% in RYGB, 40.0% in sleeve gastrectomy, and 41% in the control group. Finally, a prospective cohort study conducted by Machado et al. [19,22] investigated 58 pregnancies after previous RYGB and compared them to two groups of 58 control participants each: the first group had a BMI below 35 kg/m^2^, the second group had a BMI equal to or higher than 35 kg/m^2^. In the surgical group, the BMI at the start of pregnancy was 30 ± 6 kg/m^2^. Appropriate GWG was found in only one in four in the RYGB group (24%). Most women experienced excessive weight gain after RYGB (48.3%). Again, these findings were comparable to both control groups, where excessive GWG was seen in 41.4% and in 63.8% of the control groups with a BMI of <35 kg/m^2^ and of ≥35 kg/m^2^, respectively. 

We can, therefore, conclude that women after metabolic bariatric surgery often still have overweight or obesity in early pregnancy. In addition, these studies also indicate that GWG in these pregnancies is often not adequate, mostly because of excessive GWG according to NAM recommendations. 

## 5. Does Gestational Weight Gain Differ in Pregnancies Following Sleeve Gastrectomy versus RYGB?

In a recent systematic review aimed at evaluating which type of metabolic bariatric surgery would be most advisable for women of reproductive age, the authors concluded that there is currently insufficient evidence to provide specific recommendations [28]. 

We examined whether GWG is different in pregnancies following sleeve gastrectomy versus RYGB. Two very recent cohort studies with a non-surgical control group examined this question. In 2024, Ferreira et al. [14] conducted a retrospective observational study, looking at GWG in 63 pregnancies after RYGB and 26 pregnancies after sleeve gastrectomy. Mean weight gain in both groups was comparable: 10.74 ± 6.98 kg after RYGB and 10.20 ± 7.97 kg after sleeve gastrectomy. Next, they compared this to a control group with a BMI of ≥35 kg/m^2^. In this group, they found a mean weight gain of 7.33 ± 6.00 kg, which was significantly lower. Secondly, in the previously mentioned study conducted by Iacovou et al. in 2023 [11], GWG in pregnancies after gastric band, RYGB and sleeve gastrectomy was examined in a prospective longitudinal study. All subgroups were matched to a control group according to pre-pregnancy BMI. Both the amount and adequacy of GWG were very comparable between the three groups, with a mean weight gain of 8.57 ± 4.44 kg after RYGB, 9.10 ± 5.34 kg after sleeve gastrectomy, and 8.35 ± 5.85 kg in the control group. The authors performed a second analysis by matching all post-surgical pregnancies to a pre-surgery BMI-matched control group. In this analysis on a smaller subgroup, the GWG was significantly lower in the control group, as compared to the surgical groups with a mean weight gain of 6.21 ± 5.15 kg. This is not surprising, since the pre-pregnancy BMI in the control group was 43.19 ± 7.53. 

To conclude, according to these two recent observational studies, GWG after sleeve gastrectomy versus RYGB is similar. However, a recent systematic review and meta-analysis comparing neonatal outcomes in pregnancy after RYGB versus sleeve gastrectomy by Mustafa et al. [29] reported that while there is a reduced risk of large-for-gestational-age (LGA) neonates and gestational diabetes, there may be an increased likelihood of SGA neonates after RYGB versus sleeve gastrectomy. Certainly, additional factors may contribute to the observed outcomes, as RYGB and sleeve gastrectomy operate through distinct physiological mechanisms. 

## 6. Does the Surgery-to-Conception Interval Impact Gestational Weight Gain after Metabolic Bariatric Surgery?

Several studies have shown that there is indeed an association between a short surgery-to-conception interval and insufficient GWG; see Supplementary File S2. 

Recently, Ferreira et al. [14] compared GWG in pregnancies after RYGB and sleeve gastrectomy when pregnancy occurred at an interval smaller or larger than twelve months after surgery. In twelve out of 89 women, pregnancy occurred within one year after surgery; their mean weight gain was 4.18 ± 8.32 kg. In most women (77/89), pregnancy occurred after the first postoperative year and GWG was considerably better—at 11.73 ± 6.53 kg—which was slightly higher than the GWG of the entire cohort after metabolic bariatric surgery—10.58 ± 9.95 kg—and considerably higher than the control group—7.33 ± 6.00 kg. Looking at this issue specifically after sleeve gastrectomy, firstly, Karadağ et al. [10] conducted a retrospective cohort study examining pregnancies after sleeve gastrectomy compared to a control group with obesity (BMI > 30 kg/m^2^). A large proportion of the women in the study group became pregnant within one year after surgery (48/90), while the remaining 42 became pregnant after one year. Mean GWG was significantly lower (2.5 ± 2.9 kg) in the early group as compared to 9.5 ± 3.1 kg in the group after one year. GWG in the control group with obesity was significantly higher than in both surgical groups: 14.4 ± 3.6 kg. Secondly, Rottenstreich et al. [8,20,21] examined GWG following sleeve gastrectomy. The authors published several papers with overlapping patient populations. In the reference paper we used, GWG was compared between the group after sleeve gastrectomy versus a one-on-one matched control group. According to pre-surgical BMI, there was no significant difference in weight gain between the two groups: 10 kg in both groups (no SD given). In two subsequent papers with overlap in their study population, the authors examined the impact of the surgery-to-conception interval. In the first study, the difference between pregnancy before and 18 months post-surgery was examined [21]. The authors found insufficient GWG in one-third of the population with shorter intervals (32.8%), compared to only 13.8% in the population with longer intervals. On the other hand, half of women with longer intervals had excessive GWG (50.6%), compared to 22.4% in the short-interval group. Lastly, the same researchers examined the impact of a very short interval (<6 months) on pregnancy outcomes [20]. They identified 23 pregnancies in a very short interval; disturbingly, three women were pregnant at the time of surgery. Almost three out of four women in the very short interval experienced insufficient GWG (73.9%), with a median GWG of 4 kg (IQR −4–5 kg), despite the median pre-pregnancy BMI of 34.2 kg/m^2^ (IQR 31.2–39.4 kg/m^2^). 

Summarizing these results, a short surgery-to-conception interval is associated with insufficient GWG. On the other hand, some data indicate that a long interval may be associated with excessive GWG. 

## 7. How Does Gestational Weight Gain Impact Birth Weight Following Metabolic Bariatric Surgery?

In pregnancy outside the context of metabolic bariatric surgery, a systematic review and meta-analysis by Goldstein et al. [27] evaluated the impact of complying with NAM guidelines on pregnancy outcomes in more than one million women. They looked at the impact of insufficient or excessive GWG compared to adequate GWG for different BMI categories. As anticipated, when weight gain was below the recommended guidelines, the odds for SGA increased. However, this risk decreased as BMI increased. In other words, women with obesity in early pregnancy may have less harm from inadequate weight gain compared to women with normal weight. The reverse was seen for LGA. 

The largest population-based cohort study examining birth weight outcomes after RYGB was conducted in 2015 by Johansson et al. [2], and looked at data from the Swedish Medical Birth Register. A group of 596 pregnancies after RYGB was matched one-on-one with 2356 control pregnancies based on pre-surgery BMI, age, parity, and other factors. In the RYGB group, the mean BMI in early pregnancy was 30.6 ± 5.2, which is in the obesity range. Thus, solely based on NAM guidelines, one would expect that adherence to the guidelines would result in a low risk of SGA and that even if GWG were insufficient, a higher BMI would be protective for SGA. In addition, more macrosomia or LGA would be expected. However, the results indicated that the proportion of SGA in the metabolic bariatric surgery group was (not significantly, *p* = 0.34) higher than in the control group: 15.6% versus 7.6%, with an odds ratio of 1.39 (0.71–2.74). Furthermore, adjustment for GWG did not substantially affect the association between the surgery and this outcome. Of note, the proportion of LGA was significantly lower in the RYGB group: 6.6% versus 24.2% in the control, with an odds ratio of 0.23 (0.12–0.44), *p* < 0.001, which remained significant when adjusting for GWG. Several smaller cohort studies confirm these findings. Firstly, in a retrospective cohort study by Gascoin et al. [13], pregnancies after previous RYGB were compared to control pregnancies matched for age, parity, and smoking habits, with 56 singleton pregnancies in both groups. GWG in the RYGB group tended to be lower (11.0 [2.0; 16.0] kg) than in the control group (13.0 [10.0; 16.0] kg). Birth weight was 3000 ± 570 g and 3350 ± 430 g after RYGB and in control pregnancies, respectively. This was despite a higher preoperative BMI in the RYGB group (30.1 ± 6.0 kg/m^2^) compared to the control group (22.3 ± 4.0 kg/m^2^). Even more clinically relevant was the significant proportion of neonates born SGA after RYGB (23%) as compared to the control group (3.6%). Secondly, a retrospective observational study by Gohier et al. [30] without a control group reported on 122 pregnancies after RYGB. In that study, the average weight gain during pregnancy was 8.2 ± 6.9 kg, and 50% had insufficient GWG. In multivariate analysis, insufficient GWG was independently and significantly associated with prematurity (OR 7.8, CI 95% [1.59–38.2], *p* = 0.011). Lastly, a large retrospective cohort study by Snoek et al. [31] used multivariate regression analysis to investigate the association between birth weight and GWG in pregnancies after RYGB compared to a control group. In line with previous studies, birth weight was significantly lower in this cohort, with twofold increased odds for SGA. However, they did not find a significant association between GWG and birth weight according to gestational age. This suggests, therefore, that pregnancy after RYGB increases the risk of an SGA neonate significantly, irrespective of the amount of GWG.

Of note, when the surgery-to-conception interval is very short, birth weight becomes more clearly impacted by insufficiency in GWG. In the aforementioned study by Ferreira et al. [14], the mean birth weight percentile decreased from 34.0 ± 26.8 to 23.5 ± 30.5 when the surgery-to-conception interval was shorter than twelve months. As could be expected, this was even more so in the study by Rottenstreich et al. [20]. Here, one out of four neonates were born SGA (26.1%) when the surgery-to-conception interval was shorter than six months after sleeve gastrectomy. As mentioned previously, in this cohort, almost three out of four women experienced insufficient GWG. 

As mentioned in the previous section, some data indicate that a longer surgery-to-conception interval may be associated with an increase in excessive GWG [21]. Similar to these observations, Sancak et al. [32] performed a retrospective cohort study investigating 119 pregnancies after sleeve gastrectomy. Pregnancies were grouped according to the type of GWG: insufficient, adequate, or excessive. There was significantly more excessive GWG when the surgery-to-conception interval was longer. Interestingly, the authors found that both excessive GWG (adjusted RR = 0.54 95% CI 0.33–0.90) and higher BMI at conception (adjusted RR: 0.48, 95% CI 0.27–0.86) were independently associated with reduced risks of adverse neonatal outcomes, including SGA. This is in line with findings from Yu et al. [33], confirming that excessive versus adequate GWG decreases the odds of preterm birth (odds ratio 0.12; 95 CI 0.02–1.00). 

In summary, several studies indicate that birth weight is negatively affected by prior metabolic bariatric surgery, despite pre-pregnancy BMI being in the overweight or obesity range. Additionally, some research suggests that excessive GWG may have a protective effect on neonatal outcomes. However, these findings should be interpreted with caution, as excessive GWG can lead to an increased incidence of LGA. Moreover, it poses a risk for substantial post-pregnancy weight retention in a population already prone to obesity.

## 8. Can Dietary Interventions Improve Gestational Weight Gain after Metabolic Bariatric Surgery? 

The research in this field is very scarce. A nested case–control study by Akhter et al. [34] compared pregnancies with and without SGA in a cohort of women who underwent various types of metabolic bariatric surgery. They found that for every kilogram of GWG, there was a protective effect against SGA births, with a decreased adjusted odds ratio of 0.95 (95% CI 0.85–0.99, *p* = 0.029). Interestingly, women who reported receiving nutritional advice between surgery and pregnancy were significantly less likely to have an SGA birth, with a decreased adjusted odds ratio of 0.15 (95% CI 0.04–0.55, *p* = 0.004).

In 2022, Araki et al. [18] conducted a clinical trial investigating the effect of biweekly visits with a certified dietician on pregnancy outcomes after sleeve gastrectomy and RYGB. Because of ethical considerations, there were two control groups: one retrospective with pregnancy after surgery, the other without metabolic bariatric surgery history and without nutritional interventions. This makes the outcomes difficult to interpret. Furthermore, the number of participants was small (20 in the interventional group). The authors concluded that counseling may have contributed to normalizing GWG and improving the nutritional composition of the diet. However, this did not reflect a significant improvement in birth weight or the occurrence of SGA. Secondly, Hedderson et al. [35] conducted a retrospective analysis of the impact of telephonic nutritional management in pregnancies following metabolic bariatric surgery. GWG was evaluated according to NAM guidelines [3] and did not change by participation in the program. Nonetheless, these women were less likely to experience adverse neonatal outcomes and have micronutrient deficiencies. One of the limitations of this study was that participation was based on patient preference, which could mean that women in the intervention group were more willing to have a healthy lifestyle. Finally, Caredda et al. [36] investigated attitudes and behaviors towards nutrition and weight in pregnancy after biliopancreatic diversion and sleeve gastrectomy. The researchers observed no differences in attitudes towards weight gain; however, women in the metabolic bariatric surgery group had different eating behaviors. 

In conclusion, research on dietary interventions to improve GWG in pregnant women with prior metabolic bariatric surgery is lacking. 

## 9. Limitations of This Review

This review has some limitations. The analyses presented were selected based on their clinical relevance and the current literature. Given that this is a non-systematic review, there is an inherent selection bias in the topics discussed. For example, the impact of maternal age, socio-economic status, and behaviors such as smoking and drinking were not included. Additionally, despite the substantial amount of data collected, no further statistical analyses were conducted, nor were the authors of the original research papers contacted for missing information. 

## 10. How to Move Forward?

This scoping review reveals that women who underwent bariatric metabolic surgery often maintain overweight or obesity in early pregnancy. Nonetheless, the impact of this chronic disease intervention on pregnancy outcomes sets these pregnancies apart from those of untreated women. Healthcare professionals providing care for pregnant women after metabolic bariatric surgery should be mindful of confirmation bias. This bias may lead to an over-reliance on established guidelines on GWG for the general population, which typically address risks associated with excessive weight gain in pregnancy, potentially overlooking evidence indicating that these women are more likely to have SGA babies and, thus, may be at a higher risk of insufficient weight gain. 

Further longitudinal studies investigating the impact of GWG on maternal and neonatal outcomes after metabolic bariatric surgery are needed. For this research question, a control group with pre-pregnancy-matched BMI is most suitable. Furthermore, known confounders such as smoking habits, alcohol consumption, and maternal age should be adequately documented. Efforts should be made to report data from subgroups within surgical patient populations to ensure this information can be accurately extracted and analyzed in the future. 

Since the optimal amount of GWG for any given woman after metabolic bariatric surgery remains unclear, nutritional interventions should integrate recommendations from a registered bariatric dietician with pregnancy nutritional guidelines in a patient-tailored approach. Recognizing obesity as a chronic disease is crucial, given the persistent stigma within this patient population. Therefore, healthcare providers must be cognizant of the emotional vulnerability of these pregnant women. GWG should primarily serve as a screening measure to identify individuals requiring intensified monitoring. 

## Figures and Tables

**Table 1:** Recommended gestational weight gain based on preconception BMI.

Preconception BMI		Recommended Total Weight Gain (kg)
Underweight	<18.5 kg/m^2^	12.5–18
Normal weight	18.5–24.9 kg/m^2^	11.5–16
Overweight	25.0–29.9 kg/m^2^	7–11.5
Obesity	≥30.0 kg/m^2^	5–9

**Table 2:** Nutritional recommendations in pregnancy after metabolic bariatric surgery.

	ACOG	FIGO	SOGC	RCOG	RANZCOG
**Monitoring**	Nutritional deficiencies	Vitamin and mineral deficiencies Nutritional status Fetal growth	Inadequate nutrition	Nutritional deficiencies	Nutritional status Fetal growth
**Intervention**	Vitamin supplementation if needed	Dietician advice for nutritional needs	Maternal-fetal medicine consultant Serial growth ultrasound in 3rd Trimester	Dietician advice for nutritional needs Consultant-led care	Lifelong vitamin supplementation Dietician referral

Adapted from Giouleka et al., Table 1 [6]. ACOG: American College of Obstetricians and Gynecologists, FIGO: The International Federation of Gynecology and Obstetrics, SOGC: Society of Obstetricians and Gynaecologists of Canada, Royal College of Obstetricians and Gynaecologists, RANZCOG: Royal Australian and New Zealand College of Obstetricians and Gynaecologists.

**Table 3:** GWG according to NAM recommendations in pregnancies after metabolic bariatric surgery: cohort studies with control group.

		Hammeken 2017 [12]			Stentebjerg 2023 [9]			Machado 2020 [19]				Iacovou 2023 [11] °					
Population		RYGB	Control Pre-Pregnancy BMI ~	*p*-Value	RYGB	Control Pre-Pregnancy BMI ~	*p*-Value	RYGB	Control BMI< 35 kg/m^2^ ~	Control BMI≥ 35 kg/m^2^ ~	*p*-Value	RYGB	SG	Control Pre-Pregnancy BMI ~	RYGB	SG	Control Pre-Surgery BMI §
number =		151	151		23	23		58	58	58		49	30	100	25	13	50
Age (in years)		30.73 ± 4.72	30.69 ± 14.68	NA	35 (31–38)	30 (26–32)	**<0.01**	32 ± 5	32 ± 5	32 ± 5	0.838	34.14 ±5.39	33.90 ± 5.07	32.54 ± 5.02	33.24 ± 4.83	33.46 ± 3.75	32.54 ± 4.70
BMI (in kg/m^2^)		29.11 ± 5.33	29.03 ± 5.44	NA	32 (27–39)	33 (28–40)	0.88	30 ^a^ ± 6	25 ^b^ ± 3	39 ^c^ ± 5	**0.001**	33.08 ± 4.77	32.76 ± 4.68	33.61 ± 5.17	31.57 ± 4.70	30.27 ± 4.15	43.19 ± 7.53
Weight gain (in kg)		11.51 ± 8.97	12.18 ± 16.28	0.169	9 (2–18)	12 (7–17)	0.19	10 ^a^ (7–13)	14 ^b^ (10–19)	12 ^ab^ (8–15)	**0.007**	8.57 ± 4.44	9.10 ± 5.34	8.35 ± 5.85	8.98 ± 4.40	9.53 ± 4.79	6.21 ± 5.15
	Insufficient *	15.2% (23)	13.9% (21)	0.277	39% (9)	9% (2)	0.05	27.6% (16)	22.4% (13)	13.8% (8)	0.096	28.6% (14)	23.3% (7)	32% (32)	28.0% (7)	15.4% (2)	36.0% (18)
	Appropriate *	25.8% (39)	25.8% (39)	0.739	17% (4)	35% (8)	NA	24% (14)	36.2% (21)	22.4% (13)	NA	30.6% (15)	36.7% (11)	27% (27)	44.0% (11)	46.1% (6)	36.0% (18)
	Excessive *	39.7% (60)	46.4% (70)	0.269	44% (10)	57% (13)	NA	48.3% (28)	41.4% (24)	63.8% (37)	NA	40.8% (20)	40.0% (12)	41% (41)	28.0% (7)	38.5% (5)	28.0% (14)
	Missing *	19.2% (29)	13.9% (21)	-	-	-	-	-	-	-	-	-	-	-	-	-	-
Birth weight (in g)		3232.30 ± 619.96	3499.28 ± 595.49	NA ^	3365 (3035–3695)	3630 (3355–3920)	0.08	3078.9 ^a^ ± 430.5	3261.2 ^b^ ± 478.2	3385.4 ^b^ ± 629.4	**0.003**	3092.4 ± 496.7	3223.1 ± 483.4	3444.0 ± 486.8	3208.0 ± 493.7	3325.9 ± 555.2	3522.5 ± 525.2
Proportion SGA		10.6% (16)	4.0% (6)	**0.040**	26% (6)	4% (1)	0.10	1.7% (1)	5.2% (3)	1.7% (1)	NA	24.5% (12)	23.3% (7)	14% (14)	16% (4)	0.8% (4)	6.0% (3)
Proportion LGA		0.7% (1)	4.6% (7)	0.069	13% (3)	9% (2)	1.00	6.9% (4)	17.2% (10)	37.9% (22)	NA	4.1% (2)	6.7% (2)	15% (15)	8.0% (2)	15.4% (2)	26.0% (13)

Data are presented as mean ± SD, median (IQR), % (n), statistically significant *p*-values are in bold, NA: not available. GWG: gestational weight gain, NAM: national academy of medicine, BMI: body mass index, SGA: small-for-gestational-age, LGA: large-for-gestational-age, ~ control group matched according to pre-pregnancy BMI, § control group matched according to pre-surgery BMI, * According to NAM recommendations, ° no group-specific *p*-values were provided for this article, as *p*-values were only reported for the entire heterogeneous surgical group, ^ *p*-value for birth weight according to z-scores: 0.002. ^a, b, c, ab^ Mean, median, or proportion values followed by different letters significantly differ according to analysis of variance.

## Supplementary Materials

The following supporting information can be downloaded at https://www.mdpi.com/article/10.3390/nu16152516/s1, File S1: Search string; File S2: Overview of studies.
