# Supplementary material for: Gestational Weight Gain Following Metabolic Bariatric Surgery: A Scoping Review
Source: Nutrients. 2024 Aug 1;16(15):2516. doi: 10.3390/nu16152516 (PMC11313895; doi:10.3390/nu16152516)
Supplement: Supplementary file 1 [file nutrients-16-02516-s001.zip › File S1 search string.pdf]

1 Pubmed (via NCBI, including MEDLINE)

Concept 1: bariatric surgery

"Bariatric Surgery"[Mesh:NoExp] OR “Bariatrics”[Mesh] OR “metabolic surg*”[tiab] OR "Gastric Bypass"[Mesh] OR "Gastrectomy"[Mesh:NoExp] OR "bariatr*"[tiab] OR “gastric bypass*”[tiab] OR “sleeve gastrectom*”[tiab] OR "gastric sleeve"[tiab] OR “Roux en Y”[tiab] OR "RYGB"[tiab] OR "weight loss surgery"[tiab:~1] OR "weight loss surgical"[tiab:~1] OR "weight loss operation"[tiab:~1] OR "weight loss surgeries"[tiab:~1] OR "weight loss procedure"[tiab:~1] OR "weight loss procedures"[tiab:~1] OR "weight reduction surgery"[tiab:~1] OR "weight reduction surgical"[tiab:~1] OR "weight reduction operation”[tiab:~1] OR "weight reduction surgeries"[tiab:~1] OR "weight reduction procedure"[tiab:~1] OR "weight reduction procedures"[tiab:~1]

Concept 2: gestational weight gain

"Gestational Weight Gain"[Mesh] OR “weight gain pregnancy”[tiab:~1] OR “weight gain gestational”[tiab:~1] OR “pregnancy weight”[tiab:~1] OR “gestational weight"[tiab:~1]

#1 AND #2

2 Embase (embase.com)

Concept 1: bariatric surgery

'bariatric surgery'/de OR ‘Bariatrics’/exp OR ‘metabolic surger*’:ti,ab,kw OR 'gastric bypass surgery'/exp OR 'sleeve gastrectomy'/exp OR ‘bariatr*’:ti,ab,kw OR ‘gastric bypass*’:ti,ab,kw OR ‘sleeve gastrectom*’:ti,ab,kw OR ‘Roux en Y’:ti,ab,kw OR 'gastric sleeve':ti,ab,kw OR ‘RYGB’:ti,ab,kw OR ('weight loss' NEAR/2 ('surg*' OR 'operati*' OR 'procedure*')):ti,ab,kw OR ('weight reduction’ NEAR/2 ('surg*' OR 'operati*' OR 'procedure*')):ti,ab,kw

Concept 2: gestational weight gain

‘gestational weight gain’/de OR (‘weight gain’ NEAR/2 (‘pregn*’ OR ‘gestational’)):ti,ab,kw OR (‘weight’ NEAR/2 (‘pregn*’ OR ‘gestational’)):ti,ab,kw

Advanced mapping: OFF

#1 AND #2 NOT ‘conference abstract’:it

3 Web of Science Core Collection

Editions

Science Citation Index Expanded
(SCI-EXPANDED)--1955-present

Social Sciences Citation Index
(SSCI)--1956-present

Arts & Humanities Citation Index
(AHCI)--1975-present

Conference Proceedings Citation Index – Science
(CPCI-S)--1990-present

Conference Proceedings Citation Index – Social Science & Humanities
(CPCI-SSH)--1990-present

~~Book Citation Index – Science~~
~~(BKCI-S)--2005-present~~

Book Citation Index – Social Sciences & Humanities
(BKCI-SSH)--2005-present

Emerging Sources Citation Index
(ESCI)--2019-present

~~Current Chemical Reactions~~
~~(CCR-EXPANDED)--1985-present~~

~~Index Chemicus~~

~~(IC)--1993-present~~

<u>Concept 1: bariatric surgery</u>

TS=(“Bariatr*” OR (“bariatric” NEAR/2 (“surg*” OR “operati*” OR “procedure*”)) OR “gastric bypass*” OR ”RYGB” OR “Roux en Y” OR “metabolic surger*” OR “sleeve gastrectom*” OR “gastrectomy” OR “gastric sleeve” OR “Roux en Y” OR ("weight loss" NEAR/2 ("surg*" OR "operati*" OR "procedure*")) OR ("weight reduction" NEAR/2 ("surg*" OR "operati*" OR "procedure*")))

<u>Concept 2: gestational weight gain</u>

TS=(“gestational weight gain” OR (“weight gain” NEAR/2 (“pregn*” OR “gestational”)):ti,ab,kw OR (“weight” NEAR/2 (“pregn*” OR “gestational”)))

#1 AND #2 NOT DT=(“meeting abstract”)

4 Scopus

Concept 1: bariatric surgery

TITLE-ABS(“bariatrics” OR ("bariatric" W/2 ("surg*" OR "operati*" OR "procedure*")) OR “gastric bypass*” OR “RYGB” OR “metabolic surger*” OR “sleeve gastrectom*” OR “Roux en Y” OR ("weight loss" W/2 ("surg*" OR "operati*" OR "procedure*")) OR ("weight reduction" W/2 ("surg*" OR "operati*" OR "procedure*"))) OR AUTHKEY(“bariatrics” OR ("bariatric" W/2 ("surg*" OR "operati*" OR "procedure*")) OR “gastric bypass*” OR “RYGB” OR “metabolic surger*” OR “sleeve gastrectom*” OR “Roux en Y” OR ("weight loss" W/2 ("surg*" OR "operati*" OR "procedure*")) OR ("weight reduction" W/2 ("surg*" OR "operati*" OR "procedure*")))

Concept 2: gestational weight gain

TITLE-ABS(“gestational weight gain” OR (“weight gain” W/2 (“pregn*” OR “gestational”)) OR (“weight” W/2 (“pregn*” OR “gestational”))) OR AUTHKEY (“gestational weight gain” OR (“weight gain” W/2 (“pregn*” OR “gestational”)) OR (“weight” W/2 (“pregn*” OR “gestational”)))

#1 AND #2

5 CENTRAL (Cochrane Library)

Concept 1: bariatric surgery

#1: [mh ^"Bariatric Surgery"] OR [mh "Gastric Bypass"] OR [mh ^"Gastrectomy"]

#2: (bariatr* OR (gastric NEXT bypass*) OR (metabolic NEXT surg*) OR (sleeve NEXT gastrect*) OR “Roux en Y” OR “RYGB” OR (gastric NEXT sleeve) OR ("weight loss" NEAR/2 (surg* OR operati* OR procedure*)) OR ("weight reduction" NEAR/2 (surg* OR operati* OR procedure*))):ti,ab,kw

#3: #1 OR #2

Concept 2: gestational weight gain

#4: [mh ^"gestational weight gain"]

#5: (“weight gain” NEAR/2 (pregn* OR gestational)):ti,ab,kw

#6: (“weight” NEAR/2 (pregn* OR gestational)):ti,ab,kw

#7: #4 OR #5 OR #6

#3 AND #7
