# Supplementary material for: Gestational Weight Gain Following Metabolic Bariatric Surgery: A Scoping Review
Source: Nutrients. 2024 Aug 1;16(15):2516. doi: 10.3390/nu16152516 (PMC11313895; doi:10.3390/nu16152516)
Supplement: Supplementary file 1 [file nutrients-16-02516-s001.zip › File S2 - overview of studies.pdf]

Rottenstreich et al.

Control group: matched one-to-one for preoperative BMI

	Rottenstreich et al. 03-2018		Rottenstreich et al. 09-2018		Rottenstreich et al. 2021	
	SG	Control	SG < 18months	SG ≥ 18months	SG < 6months	SG ≥ 6months
n=	119	119	67	87	23 (3/23~)	173
Age (in years)	32 ~<br>32 (29 - 36)	31 ~<br>32 (28 - 34)	32 ~<br>32 (29-36)	32 ~<br>32 (28-36)	32 ~<br>30 (27 - 36)	32 ~<br>32 (29 - 36)
BMI at start of pregnancy (in kg/m²)	29.5 ~<br>28.9 (26.6 - 32.0)	41.5 ~<br>40.9 (38.4 - 44.8)	29.9 ~<br>29.9 (26.4 - 32.4)	29.8 ~<br>29.3 (27.3 - 31.2)	34.8 ~<br>34.2 (31.2 - 39.4)	29.9 ~<br>29.4 (27.1 - 32.0)
GWG (in kg)	10 ~<br>9 (7 - 13)	10 ~<br>10 (7 - 11)	8 ~<br>8 (6-10)	12 ~<br>11 (8-14)	-1 ~<br>4 (-4 - 5)	10.5 ~<br>10 (7 - 13)
Insufficient*	NR	NR	32.8% (22)	13.8% (12)	73.9% (17)	18.5% (32)
Appropriate*	NR	NR	44.8% (30)	35.6% (31)	17.4% (4)	38.7% (67)
Excessive*	NR	NR	22.4% (15)	50.6% (44)	8.7% (2)	42.8% (74)
Birth weight (in g)	3002 ~<br>3030 (2765 - 3262)	3352 ~<br>3398 (3028 - 1708)	3108 ~<br>3062 (2882 - 3364)	2990 ~<br>3050 (2760 - 3345)	3205 ~<br>3225 (3038- 3565)	3029 ~<br>3030 (2806 - 3295)
Proportion SGA	14.3% (17)	4.2% (5)	11.9 % (8)	14.9% (13)	26.1% (6)	10.4% (18)
Proportion LGA	1.7% (2)	19.3% (23)	3.0% (2)	3.4% (3)	8.7% (2)	2.9% (5)

Data are presented as mean ± SD, median (IQR), % (n), ~ no SD available

SG: sleeve gastrectomy, GWG: gestational weight gain, BMI: body mass index, SGA: small-for-gestational-age, LGA: large-for-gestational-age, NR: not reported, * according to NAM recommendations

Stentebjerg 2023

Control group: matched for BMI, parity, fertility treatment, smoking, alcohol consumption, former gestational diabetes, and diabetes

	RYGB	Control
n=	23	23
Age in years	35 (31-38)	30 (26-32)
BMI at start of pregnancy (in kg/m²)	32 (27 - 39)	33 (28 - 40)
GWG (in kg)	9 (2-18)	12 (7-17)
Insufficient*	39% (9)	9% (2)
Appropriate*	17% (4)	35% (8)
Excessive*	44% (10)	57% (13)
Birth weight (in g)	3365 (3035 - 3695)	3630 (3355 - 3920)
Proportion SGA	26% (6)	4% (1)
Proportion LGA	13% (3)	9% (2)

Data are presented as median (IQR), % (n)

RYGB: Roux-en-Y gastric bypass , GWG: gestational weight gain, BMI: body mass index, SGA: small-for-gestational-age, LGA: large-for-gestational-age, NR: not reported, * according to NAM recommendations

Machado 2020 & Blume 2018
Control group: matched according to maternal age, delivery year, gender.

	Machado 2020			Blume 2018		
	RYGB	Control, Pre-pregnancy BMI <35kg/m²	Control 2, Pre-pregnancy BMI ≥35kg/m²	RYGB	Control Pre-pregnancy BMI < 35 kg/m2	Control Pre-pregnancy BMI ≥ 35 kg/m2
n=	58	58	58	32	32	32
Age in years	32 ± 5	32 ± 5	32 ± 5	30 ± 5	29 ± 5	30 ± 6
BMI at start of pregnancy	30 ± 6	25 ± 3	39 ± 5	30 ± 6	25 ± 4	37 ± 2
Weight gain (in kg)	10 (7-13)	14 (10-19)	12 (8-15)	9 (6-17)	14 (11-20)	12 (8-16)
Insufficient*	27.6% (16)	22.4% (13)	13.8% (8)	21.9% (7)	18.8% (6)	12.5% (4)
Appropriate*	24% (14)	36.2% (21)	22.4% (13)	25% (8)	28.1% (9)	15.6% (5)
Excessive*	48.3% (28)	41.4% (24)	63.8% (37)	53.1% (17)	53.1% (17)	71.9% (23)
Birth weight (in g)	3078.9 ± 430.5	3261.2 ± 478.2	3385.4 ± 629.4	3044 ± 405	3331 ± 450	3344 ± 561
Proportion SGA	1.7% (1)	5.2% (3)	1.7% (1)	6.3%, (2)	3.0% (1)	3.0% (1)
Proportion LGA	6.9% (4)	17.2% (10)	37.9% (22)	6.3% (2)	21.9% (7)	34.4% (11)

Data are presented as mean ± SD, % (n)
RYGB: Roux-en-Y gastric bypass, GWG: gestational weight gain, BMI: body mass index, SGA: small-for-gestational-age, LGA: large-for-gestational-age, NR: not reported, * according to NAM recommendation

Karadağ 2020
Control group: BMI > 30 kg/m², no matching

	SG	SG<br>< 12months	SG<br>≥ 12months	Control
n=	90	48	42	54
Age (in years)	NR	30.29 ± 5.09	28.8 ± 4.72	27.48 ± 3.89
BMI at start of pregnancy	NR	32.38 ± 3.63	28.90 ± 2.84	31.05 ± 3.12
GWG (in kg)	NR	2.5 ± 2.9	9.5 ± 3.1	14.4 ± 3.6
GWG according to NAM	NR	NR	NR	NR
Birth weight (in g)	NR	NR	NR	NR
Proportion SGA	NR	22.9 % (11)	11.9% (5)	7.4% (4)
Proportion LGA	NR	4.2% (2)	4.8% (2)	14.8% (8)

Data are presented as mean ± SD, % (n)
SG: sleeve gastrectomy , GWG: gestational weight gain, BMI: body mass index, SGA: small-for-gestational-age, LGA: large-for-gestational-age, NR: not reported, NAM: national academy of medicine recommendations

Johansson 2015

Control group: matched one-on-one, based on age, parity, presurgery BMI , early pregnancy smoking status, educational level, delivery year

	Unmatched data				Matched for pre-surgery BMI			
	RYGB		Control		RYGB		Control	
n=	670	219 §	627.023	209,265§	596	179 §	2356	678 §
Age in years	31 ± 5	NR	30 ± 5		31 ± 5	NR	31 ± 5	NR
BMI at start of pregnancy	30.6 ± 5.2	NR	24.6 ± 4.6	NR	30.3 ± 4.9	NR	41.8 ± 4.8	NR
Weight gain in kg	NR	8.8 ~	NR	9.0 ~	NR	NR	NR	NR
GWG according to NAM	NR	NR	NR	NR	NR	NR	NR	NR
Birth weight in g	NR	NR	NR	NR	NR	NR	NR	NR
Proportion SGA	NR	NR	NR	NR	15.6% (92/590)°	9.3% (17)	7.6% (178/2336)°	5.8% (40)
Proportion LGA	NR	NR	NR	NR	8.6% (51/590)°	6.6% (12)	22.4% (523/2336)°	24.2% (167)

Data are presented as mean ± SD, % (n), § subgroup with data on weight gain, ~ no SD available, ° in RYGB group: data available for 590 pregnancies, in control group: data available for 2336 pregnancies

RYGB: Roux-en-Y gastric bypass, GWG: gestational weight gain, BMI: body mass index, SGA: small-for-gestational-age, LGA: large-for-gestational-age, NR: not reported, NAM: national academy of medicine recommendations

Iacovou 2023
Control: matched for age, ethnic group and parity
Additionaly, 100 were matched to early pregnancy BMI and 50 to pre-surgery BMI

	Matched pre-pregnancy BMI			Matched pre-surgery BMI		
	RYGB	SG	Control	RYGB	SG	Control
n=	49	30	100	25	13	50
Age (in years)	34.14 ±5.39	33.90 ± 5.07	32.54 ± 5.02	33.24 ± 4.83	33.46 ± 3.75	32.54 ± 4.70
BMI at start of pregnancy	33.08 ± 4.77	32.76 ± 4.68	33.61 ± 5.17	31.57 ± 4.70	30.27 ± 4.15	43.19 ± 7.53
Weight gain in kg	8.57 ± 4.44	9.10 ± 5.34	8.35 ± 5.85	8.98 ± 4.40	9.53 ± 4.79	6.21 ± 5.15
Insufficient*	28.6% (14)	23.3% (7)	32% (32)	28.0% (7)	15.4% (2)	36.0% (18)
Appropriate*	30.6% (15)	36.7% (11)	27% (27)	44.0% (11)	46.1% (6)	36.0% (18)
Excessive*	40.8% (20)	40.0% (12)	41% (41)	28.0% (7)	38.5% (5)	28.0% (14)
Birth weight (in g)	3092.43 ± 496.66	3223.10 ± 483.37	3444.03 ± 486.79	3208.00 ± 493.65	3325.92 ± 555.21	3522.54 ± 525.22
Proportion SGA	24.5% (12)	23.3% (7)	14% (14)	16% (4)	0.8% (4)	6.0% (3)
Proportion LGA	4.1% (2)	6.7% (2)	15% (15)	8.0% (2)	15.4% (2)	26.0% (13)

Data are presented as mean ± SD, % (n)
RYGB: Roux-en-Y gastric bypass, SG: sleeve gastrectomy, GWG: gestational weight gain, BMI: body mass index, SGA: small-for-gestational-age, LGA: large-for-gestational-age, NR: not reported, * according to NAM recommendation

Hammeken 2017

Control group: matched one-on-one based on pre-pregnancy BMI (1 kg/m2), age (1year), parity (nullipara or multipara), smoking (non-smoker or smoker) and time of delivery (2010–2013)

	RYGB	Control
n=	151	151
Age (in years)	30.73 ± 4.72	30.69 ± 14.68
BMI at start of pregnancy	29.11 ± 5.33	29.03 ± 5.44
Weight gain (in kg)	11.51 ± 8.97	12.18 ± 16.28
Insufficient*	15.2% (23)	13.9% (21)
Appropriate*	25.8% (39)	25.8% (39)
Excessive*	39.7% (60)	46.4% (70)
Missing	19.2% (29)	13.9% (21)
Birth weight (in g)	3232.30 ± 619.96	3499.28 ± 595.49
Proportion SGA	10.6%	4.0%
Proportion LGA	0.7%	4.6%

Data are presented as mean ± SD, % (n)

RYGB: Roux-en-Y gastric bypass, GWG: gestational weight gain, BMI: body mass index, SGA: small-for-gestational-age, LGA: large-for-gestational-age, NR: not reported, * according to NAM recommendation

Gascoin 2017

Control group: matched one-on-one for age, parity, and smoking habits

	RYGB	Control
n=	56	56
Age in years	30.5 ± 4.2	30.3 ± 6.0
BMI at start of pregnancy	30.1 ± 6.0	22.3 ± 4.0
Weight gain in kg	11.0 (2.0-16.0)	13.0 (10.0-16.0)
GWG according to NAM	NR	NR
Birth weight in g	3000 ± 570	3350 ± 430
Proportion SGA	23%	3.6%
Proportion LGA	NR	NR

Data are presented as mean ± SD, median (IQR), % (n)

RYGB: Roux-en-Y gastric bypass, GWG: gestational weight gain, BMI: body mass index, SGA: small-for-gestational-age, LGA: large-for-gestational-age, NR: not reported, NAM: national academy of medicine recommendations

Ferreira 2024

Control group: not matched

	RYGB	SG	RYGB + SG	Control, BMI ≥ 35	RYGB + SG < 12mo	RYGB + SG ≥ 12mo	GWG < 10kg	GWG ≥ 10kg
n=	63	26	89	176	12	77	43	41
Age (in years)	NR	NR	32.9 ± 4.5	31.3 ± 15.7	NR	NR	NR	NR
BMI at start of pregnancy	NR	NR	30.0 ± 7.5	38.3 ± 5.3	NR	NR	NR	NR
Weight gain (in kg)	10.74 ± 6.98	10.20 ± 7.97	10.58 ± 9.95	7.33 ± 6.00	4.18 ± 8.32	11.73 ± 6.53	NR	NR
GWG according to NAM	NR	NR	NR	NR	NR	NR	NR	NR
Birth weight in percentile	35.10 ± 20.69	31.30 ± 20.86	34.24 ± 21.09	48.77 ± 27.94	23.5 ± 30.5	34.0 ± 26.8	30.42 ± 21.01	37.68 ± 19.83
Proportion SGA	12.9%, (8)	11.5%, (3)	12.5%, (11)	7%, (12)	16.7%, (2)	11.8%, (9)	2.3%, (1)	2.4%, (1)
Proportion LGA	3.2%, (2)	0%	2.3%, (2)	6.4%, (11)	8.3%, (1)	1.3%, (1)	16.3%, (7)	7.3%, (3)

Data are presented as mean ± SD, % (n)

RYGB: Roux-en-Y gastric bypass, SG: sleeve gastrectomy, GWG: gestational weight gain, BMI: body mass index, SGA: small-for-gestational-age, LGA: large-for-gestational-age, NR: not reported, NAM: national academy of medicine recommendations

de Alencar Costa 2015

Control group: not matched, women with obesity

	RYGB	Control
n=	84	73
Age (in years)	?	?
BMI at start of pregnancy	26.5±4.2	34.6±3.3
Weight gain (in kg)	9.2 ± 7.9	14.4 ± 5.4
GWG according to NAM	NR	NR
Birth weight (in g)	3172 ± 547.4	3406 ± 662.9
Proportion SGA	NR	NR
Proportion LGA	NR	NR

Data are presented as mean ± SD, % (n)

RYGB: Roux-en-Y gastric bypass, GWG: gestational weight gain, BMI: body mass index, SGA: small-for-gestational-age, LGA: large-for-gestational-age, NR: not reported, NAM: academy of medicine recommendations

Chevrot 2016

Control group: matched for prepregnancy BMI, and when possible, maternal age (<20, 20-35, and >35), ethnic origin, and parity (nulliparous or parous).

	RYGB	Control
n=	58	139
Age in years	32 ± 4.9	31.9 ± 4.7
BMI at start of pregnancy	33.1 ± 6.2	33.8 ± 5.9
Weight gain in kg	6.8 ± 6.7	8.4 ± 6.8
GWG according to NAM	NR	NR
Birth weight in g	3093 ± 452	3493 ± 479
Proportion SGA	29% (17)	6% (8)
Proportion LGA	5% (3)	17% (24)

Data are presented as mean ± SD, % (n)

RYGB: Roux-en-Y gastric bypass, GWG: gestational weight gain, BMI: body mass index, SGA: small-for-gestational-age, LGA: large-for-gestational-age, NR: not reported, NAM: academy of medicine recommendations

Carlsen 2020

Control group: not mached (80 normal weight, 231 with obesity)

	RYGB	Control
n=	25	311 (80 + 231)
Age (in years)	30.3 ± 4.1	31.2 ± 4.6
BMI at start of pregnancy	28.8 ± 14.8	30.9 ± 26
Weight gain (in kg)	13.2 ± 8.3	11.4 ± 6.1
GWG according to NAM	NR	NR
Birth weight (in g)	3284 ± 327	3619 ± 523
Proportion SGA	4% (1)	4% (13)
Proportion LGA	0% (0)	7% (22)

Data are presented as mean ± SD, % (n)

RYGB: Roux-en-Y gastric bypass, GWG: gestational weight gain, BMI: body mass index, SGA: small-for-gestational-age, LGA: large-for-gestational-age, NR: not reported, NAM: academy of medicine recommendations

Araki 2022

Control group: not mached

	Bariatric group	Non-surgical control	Retrospective bariatric control
n=	20	23	18
Age (in years)	31.75 ± 5.07	29.35 ± 4.66	33.72 ± 4.84
BMI at start of pregnancy	27.10 ± 3.22	23.32 ± 3.08	31.01 ± 7.79
Weight gain (in kg)	7.5 ± 6.5	10.3 ± 4.1	3.8 ± 12.8
GWG according to NAM	NR	NR	NR
Birth weight (in g)	3168 ± 412	3398 ± 502	3074 ± 368
Proportion SGA	6.7%	4.3%	23.5%
Proportion LGA	NR	NR	NR

Data are presented as mean ± SD, % (n)

GWG: gestational weight gain, BMI: body mass index, SGA: small-for-gestational-age, LGA: large-for-gestational-age, NR: not reported, NAM: academy of medicine recommendations
